# Quantitative MRI detects delayed perfusion and impact of bronchial artery dilatation on pulmonary circulation in patients with cystic fibrosis

**DOI:** 10.1007/s00330-025-11589-y

**Published:** 2025-04-16

**Authors:** Patricia Leutz-Schmidt, Julian Grolig, Lena Wucherpfennig, Olaf Sommerburg, Monika Eichinger, Sabine Wege, Simon Y. Graeber, Jens-Peter Schenk, Abdulsattar Alrajab, Hans-Ulrich Kauczor, Mirjam Stahl, Marcus A. Mall, Arnd Koeppe, Britta Nestler, Michael Selzer, Simon M. F. Triphan, Mark O. Wielpütz

**Affiliations:** 1 Diagnostic and Interventional Radiology, University Hospital of Heidelberg, Heidelberg, Germany; 2 Translational Lung Research Center Heidelberg (TLRC), German Center for Lung Research (DZL), Heidelberg, Germany; 3 Department of Diagnostic and Interventional Radiology with Nuclear Medicine, Thoraxklinik at the University Hospital of Heidelberg, Heidelberg, Germany; 4 Institute for Applied Materials - Microstructure Modelling and Simulation (IAM-MMS), Karlsruhe Institute of Technology (KIT), Karlsruhe, Germany; 5 Institute of Nanotechnology (INT), Karlsruhe Institute of Technology (KIT), Eggenstein-Leopoldshafen, Germany; 6 Division of Pediatric Pulmonology & Allergy and Cystic Fibrosis Center, Department of Pediatrics, University of Heidelberg, Heidelberg, Germany; 7 Department of Translational Pulmonology, University Hospital Heidelberg, Heidelberg, Germany; 8 Department of Pulmonology and Respiratory Medicine, Thoraxklinik at the University Hospital of Heidelberg, Heidelberg, Germany; 9 Department of Pediatric Respiratory Medicine, Immunology and Intensive Care Medicine, Charité-Universitätsmedizin Berlin, Berlin, Germany; 10 German Center for Lung Research (DZL) associated partner site, Berlin, Germany; 11 German Center for Child and Adolescent Health (DZKJ) partner site, Berlin, Germany; 12 Institute of Digital Materials Science (IDM), Karlsruhe University of Applied Sciences, Karlsruhe, Germany; 13 Department of Diagnostic Radiology and Neuroradiology, University Medicine Greifswald, Greifswald, Germany

**Keywords:** Bronchial arteries, Magnetic resonance imaging, Cystic fibrosis, Hemoptysis, Dynamic contrast enhanced magnetic resonance imaging

## Abstract

**Objectives:**

MRI detects abnormal lung perfusion in patients with cystic fibrosis (CF). However, little is known about the contribution of bronchial arteries to lung perfusion in CF. We hypothesized that delayed perfusion can be detected by dynamic contrast-enhanced (DCE-)MRI and that bronchial artery dilatation (BAD) is associated with changes in lung perfusion.

**Materials and methods:**

Morpho-functional MRI was prospectively acquired in 75 patients with CF (18.7 ± 7.6 years, range 6–39 years). Lungs and perfusion defects were segmented automatically to quantify perfusion defects in percent (QDP). Pulmonary blood flow (PBF), mean transit time (MTT), and perfusion delay were calculated for the whole lung, inside normally perfused and perfusion defect areas. Chest MRI score and BAD were assessed visually.

**Results:**

QDP and PBF correlated with MRI global score (*r* = 0.58 and −0.53, *p* < 0.001). In normally perfused lung, PBF was higher (161.2 ± 77.9 mL/100 mL/min vs. 57.5 ± 26.4 mL/100 mL/min, *p* < 0.001), and MTT (5.4 ± 1.7 s vs. 6.9 ± 2.3 s, *p* < 0.001) and perfusion delay were shorter than in perfusion defect areas (4.6 ± 5.3 s vs. 13.4 ± 16.2 s, *p* < 0.001). 48 (64.0%) patients showed BAD, had higher QDP (44.6 ± 20.8% vs. 17.3 ± 11.0%, *p* < 0.001) and lower PBF (91.9 ± 54.8 mL/100 mL/min vs. 178.3 ± 77.4 mL/100 mL/min, *p* < 0.001) than patients without BAD. MTT was shorter (6.3 ± 1.9 s vs. 8.0 ± 2.6 s, *p* < 0.001), and perfusion delay was longer (13.8 ± 10.1 s vs. 12.8 ± 23.7 s, *p* < 0.02) inside perfusion defects of patients with BAD compared to without BAD.

**Conclusion:**

Perfusion parameters correlate with lung disease severity, and perfusion defects showed delayed perfusion in patients with CF. BAD was associated with more extensive perfusion defects and reduced PBF.

**Key Points:**

***Question*** *Dilated bronchial arteries are a common comorbidity in cystic fibrosis (CF), which can cause hemoptysis, but their quantitative contribution to lung perfusion is little researched*.

***Findings*** *Perfusion defects in percent (QDP) enabled objective assessment of perfusion abnormalities in CF patients, while perfusion delay and arterial correlation showed bronchial artery perfusion contribution*.

***Clinical relevance*** *The usage of quantitative perfusion metrics in CF may help tracking disease progression. By also including the proposed metrics perfusion delay and arterial correlation, bronchial artery inflow could be assessed and used to detect early onset of bronchial artery dilation*.

**Graphical Abstract:**

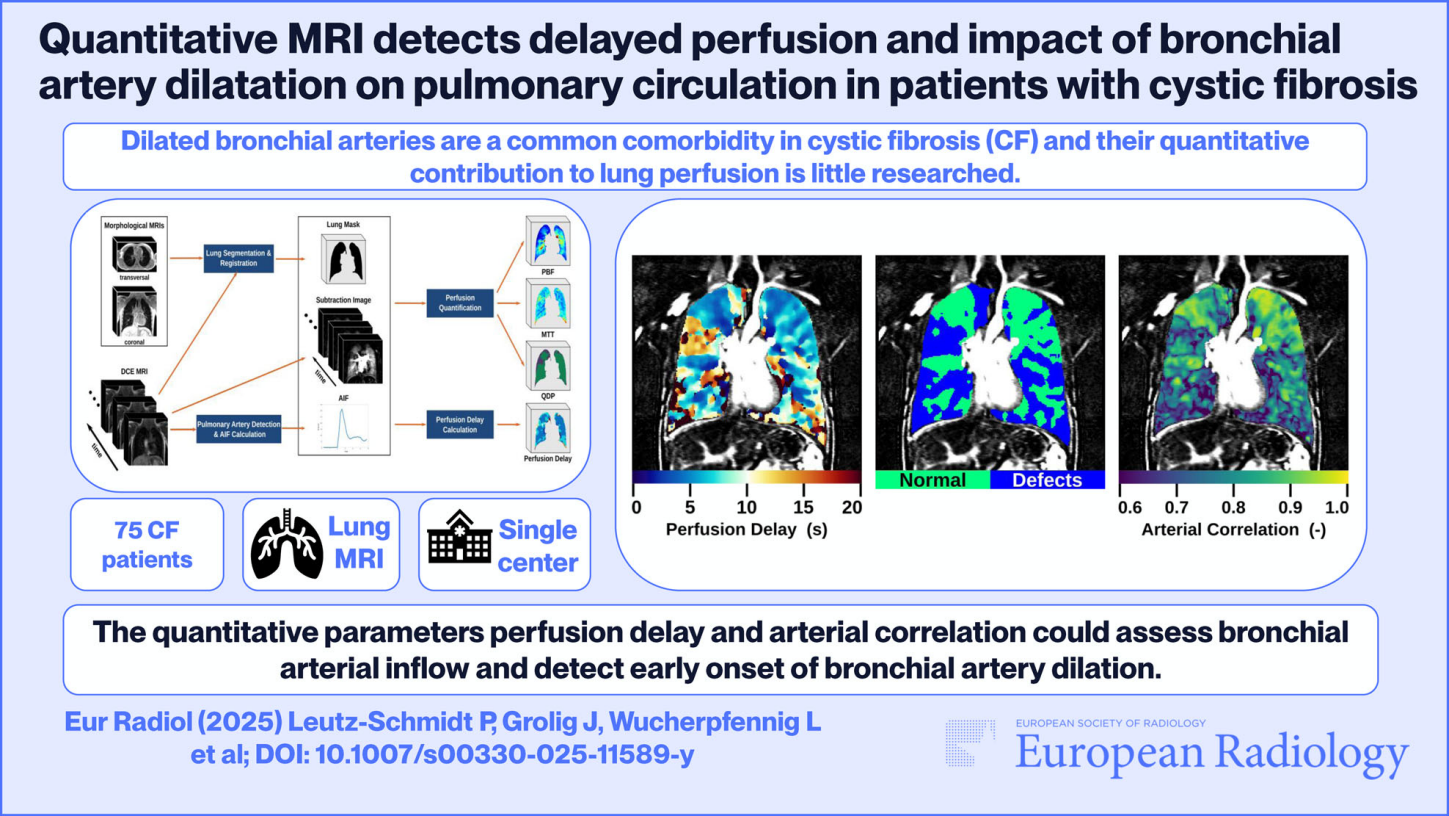

## Introduction

In patients with cystic fibrosis (CF), chronically progressive lung damage is the major cause of morbidity and mortality [1–3]. Bronchial artery dilatation (BAD) is often diagnosed late at the time of pulmonary hemorrhage experienced by 9.1% of all patients with CF during lifetime [4, 5]. Bronchial arteries are normally < 2 mm in diameter, arise from the descending aorta, and deliver approx. 1% of the left cardiac output to the lungs [6, 7]. The presence of BAD may indicate increased bronchial arterial inflow in advanced CF lung disease, driven by regional hypoxia and chronic inflammation [4, 8]. Recently, we showed that chest magnetic resonance imaging (MRI) detects BAD in patients with CF and that the occurrence of BAD is associated with more severe lung disease [9–13]. In two independent observational studies, we demonstrated that therapy with the highly effective cystic fibrosis transmembrane conductance regulator modulator (CFTRm) elexacaftor/tezacaftor/ivacaftor (ETI) reduces the chest MRI morphology score but not the MRI perfusion score in adolescents and adults with CF [14, 15]. It was shown in 6–11 years old CF patients that the MRI perfusion score improves after ETI therapy [16] demonstrating a reversibility of perfusion abnormalities in school-age children in comparison to adolescents and adults with more advanced and irreversible disease components, which was also hypothesized in other studies before [10, 17]. In contrast, another study showed that by using the quantitative phase-resolved functional lung (PREFUL) MRI approach, a quantitative improvement of perfusion after ETI therapy can even be shown in adults [18].

Further, the diameter of bronchial arteries is reduced by ETI therapy in adults with CF, which indicates an influence of ETI on pulmonary hemodynamics probably not captured by the MRI perfusion score [15]. In another study, a diameter reduction of the bronchial arteries after highly effective CFTR modulator was observed, whereas pulmonary perfusion defects did not change in adults [19]. The presence of BAD may thus be related to remodeling of pulmonary perfusion [13]. Yet, little is known about the quantitative contribution of the bronchial arteries to pulmonary perfusion in CF, especially after the onset of BAD.

Pulmonary blood flow (PBF), pulmonary blood volume (PBV) and mean transit time (MTT), and, more recently, perfusion defects in percent (QDP) can be derived from dynamic contrast-enhanced perfusion MRI (DCE-MRI) and are putative metrics to quantitatively describe pulmonary perfusion [20, 21]. DCE-MRI could potentially separate pulmonary arterial from bronchial arterial inflow into the lungs based on the time course of contrast enhancement after bolus injection, whereas QDP and the MRI perfusion score assess pulmonary arterial perfusion only [22]. Thus, we hypothesized that quantitative assessment of DCE-MRI can detect delayed perfusion related to bronchial artery perfusion as compared to pulmonary arterial perfusion. This delay may be ‘unmasked’ inside pulmonary arterial perfusion defect areas in patients with CF. Further, we assessed differences in pulmonary perfusion metrics in patients after the onset of BAD as compared to patients without BAD.

## Materials and methods

### Subjects

This study is part of an ongoing prospective observational study (clinicaltrials.gov identifiers NCT00760071, NCT02270476 and DRKS00031784) and was approved by the institutional ethics committee. Written informed consent was obtained from all patients or their parents or legal guardians. School-age and adult CF patients underwent chest MRI, spirometry, multiple-breath washout (MBW) and clinical assessment as part of their annual surveillance visit in stable clinical condition (Table 1, Supplementary Table E1) [10, 11, 23]. Some patients were included in our previous reports but without quantitatively assessing lung perfusion [11, 13]. Further details are provided in the online supplement.

**Table 1:** Characteristics of patients with cystic fibrosis without or with bronchial artery dilatation (BAD) at the timepoint of magnetic resonance imaging (MRI)

	Total	No BAD	BAD
*n*	75	27	48
Age (years) (range)	18.7 ± 7.6 (6–39)	12.7 ± 3.7 (6–18)	22.1 ± 7.2 (7–39)***
Male/female	45/30	15/12	30/18
Height (cm)	161.1 ± 16.1	153.3 ± 19.9	165.4 ± 11.4*
Height, SDS^†^	0.0 ± 1.2	0.0 ± 1.1	−0.1 ± 1.3
Weight (kg)	50.9 ± 15.4	44.0 ± 17.1	54.7 ± 12.9*
Weight, SDS^†^	−0.6 ± 0.9	−0.5 ± 0.8	−0.8 ± 1.0
BMI (kg/m^2^)^♦^	20.6 ± 2.9	21.4 ± 0.0	20.6 ± 3.0
BMI, SDS^†^	−0.7 ± 0.8	−0.7 ± 0.8	−0.9 ± 0.8
*CFTR* genotype, % (*n*)	99 (74)	100 (27)	98 (47)
F508del/F508del	47 (34)	58 (15)	40 (19)
F508del/other	47 (34)	37 (10)	51 (24)
Other/other	7 (5)	4 (1)	9 (4)
Pancreatic insufficiency, % (*n*)	89 (67/75)	85 (23/27)	92 (44/48)
*P. aeruginosa* positive, % (*n*)	45 (27/60)	15 (4/26)	68 (23/34)*
Spirometry, *n*	64	27	37
ppFEV_1_	73.0 ± 18.3	84.8 ± 16.0	64.4 ± 14.8***
Multiple-breath washout, *n*	24	21	3
LCI2%	8.5 ± 2.4	8.2 ± 2.2	10.7 ± 2.8
MRI morphology score	14.0 ± 12.7	8.0 ± 3.8	17.5 ± 9.5***
MRI perfusion score	6.0 ± 3.0	5.0 ± 3.8	7.5 ± 3.0***
MRI global score	22.0 ± 16.0	13.0 ± 5.8	25.0 ± 12.0***
QDP (%)	34.8 ± 22.2	17.3 ± 11.0	44.6 ± 20.8***
PBF (mL/100 mL/min)	123.0 ± 75.9	178.3 ± 77.4	91.9 ± 54.8***
MTT (s)	5.7 ± 1.7	6.2 ± 2.2	5.5 ± 1.2
Perfusion delay (s)	6.8 ± 7.2	5.2 ± 5.7	7.8 ± 7.8**
Arterial correlation (-)	0.87 ± 0.08	0.91 ± 0.06	0.85 ± 0.08***

Data presented as percentage (proportion), mean ± SD or median ± interquartile range for MRI scores

*BMI* body mass index, *CFTR* cystic fibrosis transmembrane conductance regulator, *ppFEV1* forced expiratory volume in 1 s in percent predicted, *LCI* lung clearance index, *P. aeruginosa* chronic *Pseudomonas aeruginosa* infection, *QDP* perfusion defects in percent, *PBF* pulmonary blood flow, *MTT* mean transit time

* *p* < 0.05, ** *p* < 0.01, and *** *p* < 0.001 vs. no BAD, respectively

^†^ SDS given for patients ≤18 years only

^♦^ BMI given for patients > 18 years only

### Morpho-functional magnetic resonance imaging

Chest MRI was acquired on clinical 1.5-T MR scanners (Magnetom Avanto and Aera, Siemens Healthineers) as previously described [9, 11–15, 24]. Further details are provided in the online supplement.

### Magnetic resonance imaging assessment

Structural and functional lung abnormalities were assessed using the validated MRI scoring system by a reader with more than 15 years of experience in chest MRI (MOW) as previously described [9–11, 24, 25]. The presence of BAD was assessed in the 4D perfusion MRI at the timepoint of maximal aorta enhancement by this reader in consensus with a radiologist with more than seven years of experience in chest MRI (PLS). Since undilated bronchial arteries would not be visible at this resolution, any artery in a typical anatomical position for a bronchial artery with a tortuous and unequivocal course along the main stem bronchi into the right or left lung hilus was considered as a positive finding for BAD as previously described [13].

For quantitative assessment, the lung was automatically segmented on the morphological images from 3D gradient echo acquisitions and registered onto DCE-MRI with a non-rigid registration using elastix [21, 26–28]. The pulmonary artery was automatically detected on DCE-MRI to calculate the arterial input function (AIF), which was then used to calculate the residual function R(t) for every voxel using deconvolution. From R(t), pulmonary blood flow (PBF) and mean transit time (MTT) were quantified [20, 21]. At every timepoint, the average value of R(t) over all lung voxels was calculated to determine the timepoint of maximum contrast enhancement. Otsu’s clustering method was then used on median-filtered R(t) maps at this timepoint to determine two thresholds for clustering the voxels into poorly perfused, well-perfused and vessel areas. Perfusion defects in percent (QDP) was defined as the relative amount of voxels in the poorly perfused cluster [21, 29]. Thus, lung voxels could be separated into normally perfused lung and perfusion defects.

As a next step, we sought to detect bronchial arterial inflow into the lungs based on the assumption that an i.v. contrast bolus passage during DCE-MRI will first pass the pulmonary arteries, which is the phase used to assess perfusion abnormalities in CF, and will pass the lungs shortly later through bronchial arteries originating from the aorta [10, 13]. Perfusion delay maps and maps for the cross-correlation between the AIF and the signal in each voxel were generated by calculating the cross-correlation between time-interpolated subtraction images and a moving window of the edge-padded AIF. For the following, the window position with the highest cross-correlation was defined as the local perfusion delay and the strength of this cross-correlation as arterial correlation. The quantification pipeline is detailed in the online supplement (Supplementary Fig. E1).

### Statistical analyses

Data were analyzed using MATLAB R2023a (The MathWorks Inc.). Data are presented as mean ± standard deviation unless otherwise specified. Median values were calculated for normally perfused lung and perfusion defect areas. An inter-patient average was then calculated using the mean of these median values, except for the visual MRI scores were the median and the interquartile range were calculated for sum scores. Quantitative perfusion parameters were compared inside areas with normal perfusion to areas representing perfusion defects, and also between patients with and without BAD using the Wilcoxon signed-rank or rank sum test as appropriate. The correlations of quantitative perfusion parameters with the MRI score and lung function tests were calculated using Spearman correlation. Averaged histograms were generated for each perfusion parameter, and the bootstrap method was used to test for significant differences, as suggested by Xu et al [30]. A *p*-value < 0.05 was considered statistically significant.

## Results

### Quantitative perfusion parameters correlate with chest MRI scores, spirometry and multiple-breath washout in patients with CF

**Fig. 1:**
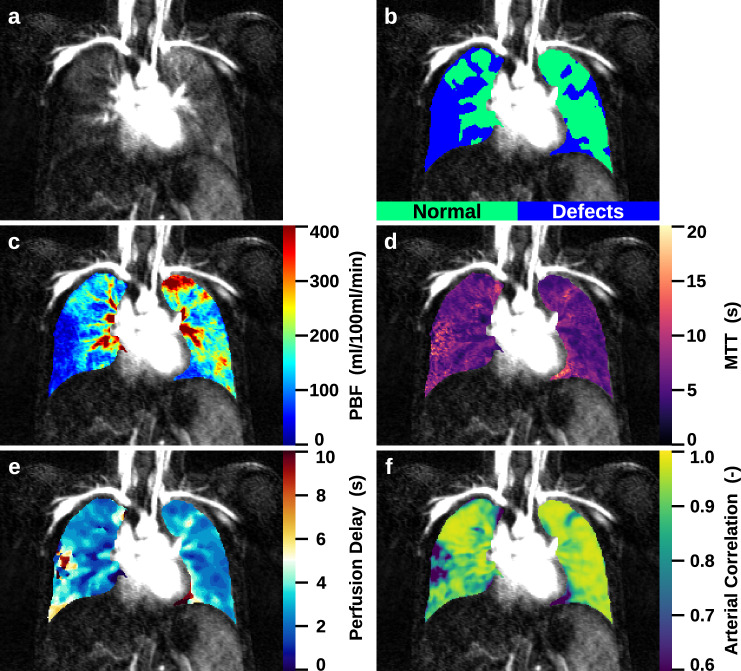
Quantitative perfusion parameter maps of a 13-year-old male CF patient without dilated bronchial arteries. **a** Subtracted perfusion map, (**b**) perfusion defects map, (**c**) PBF map, (**d**) MTT map, (**e**) perfusion delay map and (**f**) arterial correlation map. In blue are marked perfusion defects (**b**), which show lower PBF (**c**), a higher MTT (**d**), longer perfusion delays inside the defect areas (**e**) and smaller arterial correlations (**f**)

A group of 75 patients (mean age 18.7 ± 7.6 years, range 6.1–40.0 years) was included with ppFEV_1_ = 73.0 ± 18.3%. The mean MRI morphology, MRI perfusion and MRI global score were 14.0 ± 12.7, 6.0 ± 3.0, and 22.0 ± 16.0, respectively. Average QDP was 34.8 ± 22.2% (Fig. 1, Table 1). QDP and PBF correlated moderately with the MRI global (*r* = 0.66 and −0.59, *p* < 0.001), the MRI morphology score (*r* = 0.66 and −0.56, *p* < 0.001), and the MRI perfusion score (*r* = 0.54 and −0.56, *p* < 0.001), respectively. Correlation with ppFEV_1_ was moderate for QDP and PBF (*r* = −0.48 and 0.45, *p* < 0.001), and weak for perfusion delay (*r* = −0.30, *p* < 0.05) (Table 2). Median perfusion delay correlated weakly with the MRI scores (*r* = 0.49, 0.46 and 0.50, respectively, *p* < 0.001).

**Table 2:** Spearman correlations of quantitative perfusion parameters with the magnetic resonance imaging (MRI) morphology, MRI perfusion, and MRI global sore, as well as forced expiratory volume in 1 s in percent predicted and lung clearance index in patients with cystic fibrosis

	QDP	PBF	MTT	Perfusion delay
MRI morphology score, *r*	0.66***	−0.56***	−0.11	0.46***
|95% CI|	0.53, 0.75	−0.68, −0.41	−0.29, 0.09	0.29, 0.60
MRI perfusion score, *r*	0.54***	−0.56***	−0.09	0.50***
|95% CI|	0.38, 0.66	−0.68, −0.41	−0.28, 0.10	0.34, 0.63
MRI global score, *r*	0.66***	−0.59***	−0.12	0.49***
|95% CI|	0.53, 0.75	−0.70, −0.45	−0.31, 0.07	0.33, 0.62
ppFEV_1_	−0.48***	0.45***	0.07	−0.30*
|95% CI|	−0.62, −0.30	0.27, 0.60	−0.14, 0.27	−0.47, −0.09
LCI	0.20	−0.33	0.20	0.36
|95% CI|	−0.15, 0.51	−0.61, 0.01	−0.15, 0.51	0.02, 0.63

*QDP* perfusion defects in percent, *PBF* pulmonary blood flow, *MTT* mean transit time, *ppFEV*_*1*_ forced expiratory volume in 1 s in percent predicted, *LCI* lung clearance index

* *p* < 0.05, and *** *p* < 0.001

### MRI detects delayed perfusion inside perfusion defects in patients with CF

**Fig. 2:**
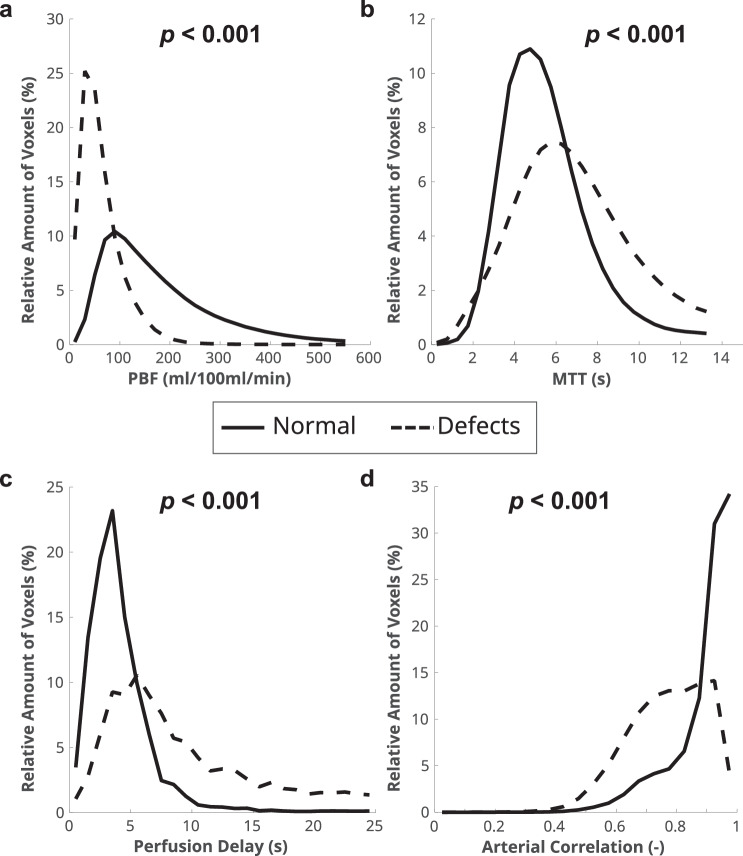
Mean histograms of normal perfused areas (solid line) and perfusion defects (dashed line) for (**a**) PBF, (**b**) MTT, (**c**) perfusion delay and (**d**) arterial correlation. The mean histograms were generated by averaging the individual histograms of all patients

Expectedly, PBF in perfusion defect areas was lower with 57.5 ± 26.4 mL/100 mL/min compared to 161.2 ± 77.9 mL/100 mL/min in normally perfused lung (*p* < 0.001). Perfusion defect areas showed longer MTT with 6.9 ± 2.3 s compared to 5.4 ± 1.7 s in normally perfused lung (*p* < 0.001). Further, perfusion delay was longer with 13.4 ± 16.2 s vs. 4.6 ± 5.3 s (*p* < 0.001), and median arterial correlation was weaker with 0.8 ± 0.1 vs. 0.9 ± 0.1 (*p* < 0.001) inside perfusion defects compared to normally perfused lung, respectively (Fig. 2, Table 3). The majority of voxels in defect areas have PBF below 100 mL/100 mL/min. For both MTT and perfusion delay, the defect histogram is wider and shifted toward higher values and predominantly shows lower arterial correlations compared to normally perfused lung (Fig. 2).

**Table 3:** Quantitative perfusion parameters in patients with cystic fibrosis without and with bronchial artery dilatation (BAD) inside normally perfused and perfusion defect areas

	Normally perfused			Perfusion defects		
	Total	No BAD	BAD	Total	No BAD	BAD
PBF (mL/100 mL/min)	161.2 ± 77.9	198.5 ± 80.7	140.2 ± 68.6***	57.5 ± 26.4^###^	75.1 ± 27.0	47.7 ± 20.5***
MTT (s)	5.4 ± 1.7	6.0 ± 2.1	5.1 ± 1.2*	6.9 ± 2.3^###^	8.0 ± 2.6	6.3 ± 1.9***
Perfusion delay (s)	4.6 ± 5.3	4.2 ± 2.5	4.9 ± 6.4	13.4 ± 16.2^###^	12.8 ± 23.7	13.8 ± 10.1*
Arterial correlation (-)	0.91 ± 0.07	0.92 ± 0.06	0.90 ± 0.07	0.78 ± 0.07^###^	0.80 ± 0.08	0.77 ± 0.06

Data presented as mean ± standard deviation

*PBF* pulmonary blood flow, *MTT* mean transit time

* *p* < 0.05, and *** *p* < 0.001 vs. No BAD, ^###^*p* < 0.001 vs. normally perfused total, respectively

### Bronchial artery dilatation is associated with altered quantitative perfusion parameters in patients with CF

**Fig. 3:**
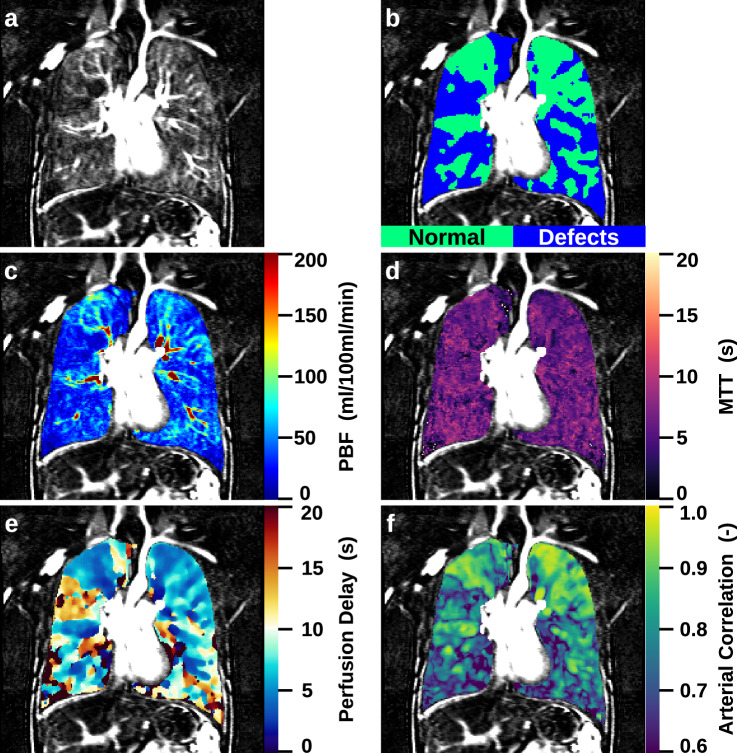
Quantitative perfusion parameter maps of a 23-year-old male CF patient with dilated bronchial arteries. **a** Subtracted perfusion map, **b** perfusion defects map, **c** PBF map, **d** MTT map, **e** perfusion delay map and **f** arterial correlation map

In 48 of 75 (64%) CF patients BAD was detected on MRI, and these patients were older (22.1 ± 7.2 years vs. 12.7 ± 3.7 years, *p* < 0.001), had lower ppFEV_1_ (64.4 ± 14.8 vs. 84.8 ± 16.0, *p* < 0.001), and a higher MRI global score (25.0 ± 12.0 vs. 13.0 ± 5.8, *p* < 0.001) compared to patients without BAD, respectively (Fig. 3, Table 1).

Patients with BAD had higher QDP compared to patients without BAD (44.6 ± 20.8 vs. 17.3 ± 11.0, *p* < 0.001), had lower whole-lung PBF (91.9 ± 54.8 mL/100 mL/min vs. 178.3 ± 77.4 mL/100 mL/min, *p* < 0.001), and tended to have a shorter MTT (5.5 ± 1.2 s vs. 6.2 ± 2.2 s, *p* = 0.056). Median perfusion delay was higher (7.8 ± 7.8 s vs. 5.2 ± 5.7 s, *p* < 0.01), and median arterial correlation was lower in patients with BAD compared to patients without BAD (0.85 ± 0.08 vs. 0.91 ± 0.06, *p* < 0.001) (Table 1). We also compared patients with and without BAD in an age-matched sub-cohort, which did not yield substantially different results and is limited by a smaller sample size (Supplementary Table E2).

**Fig. 4:**
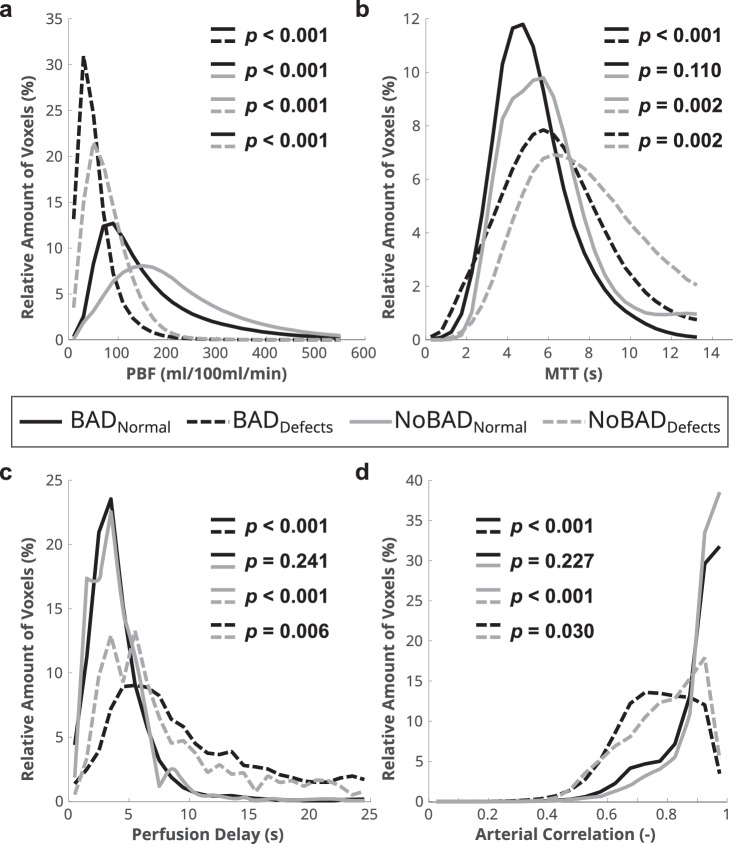
Mean histograms of normal perfused areas in BAD patients (solid black line), defect perfusion areas (dashed black line), normal perfused areas in NoBAD patients (solid gray line) and defect perfusion areas in NoBAD patients (dashed gray line) for (**a**) PBF, (**b**) MTT, (**c**) perfusion delay and (**d**) arterial correlation. The mean histograms were generated by averaging the individual histograms of all patients

When considering perfusion defect areas only, PBF was lower in CF patients with BAD compared to patients without BAD (47.7 ± 20.5 mL/100 mL/min vs. 75.1 ± 27.0 mL/100 mL/min, *p* < 0.001), MTT was shorter (6.3 ± 1.9 s vs. 8.0 ± 2.6 s, *p* < 0.001), and perfusion delay was longer (13.8 ± 10.1 s vs. 12.8 ± 23.7 s, *p* < 0.02), respectively. Median arterial correlation inside perfusion defects did not differ between patients with and without BAD (Table 3). Further, histogram analyses for perfusion delay show a shift toward longer delay (*p* < 0.01) and a wider shift toward lower arterial correlations for the defect areas in patients with BAD (*p* < 0.05) (Fig. 4). Considering normally perfused lung areas only, PBF was also lower in patients with BAD compared to patients without (140.2 ± 68.6 mL/100 mL/min vs. 198.5 ± 80.7 mL/100 mL/min, *p* < 0.001) whereas other parameters were not significantly different (Table 3).

## Discussion

With the present study, we show that the quantitative perfusion metrics QDP and PBF correlate with CF lung disease severity as measured by the CF MRI scoring system. Further, we demonstrate that QDP and PBF correlate moderately with lung function impairment measured by spirometry (Table 2). By separating normally perfused lung from areas with perfusion defects, we show that inside perfusion defect areas, a delayed perfusion can be detected in comparison to normally perfused lung (Fig. 2, Table 3).

Next, we investigated the potential influence of BAD and show that the presence of BAD was associated with worse MRI morphology, MRI perfusion and MRI global scores as well as higher QDP and lower whole-lung PBF compared to CF patients without BAD. Lastly, by comparing quantitative perfusion metrics in perfusion defects in patients with and without BAD, we found that MTT was shorter and perfusion delay even longer in CF patients with BAD compared to patients without (Fig. 4, Table 3).

In our previous works, we suggested QDP as a novel metric for the quantification of perfusion defects in patients with chronic obstructive pulmonary disease (COPD) and validated the mid-term reproducibility in adults with CF and COPD, which was better than for the traditional metric PBF [12, 21, 29]. With the present work we extend to the use of QDP as a robust metric for perfusion quantification in school-age and adolescent patients with CF. In our cohort with CF patients over a wide spectrum of ages and disease severities, we now show that QDP and PBF correlate moderately with the MRI morphology, MRI perfusion and MRI global score, as well as with ppFEV_1_, which compares favorably with previous results in adults with CF and COPD (Table 2) [20, 21]. These results support the use of QDP as an objective user-independent metric for assessing perfusion abnormalities in patients with CF from school age to adulthood. Based on the aforementioned results, we are now able to label lung voxels as belonging to normally perfused lung and perfusion defects.

A potential reason for not having stronger correlations between QDP and the visual MRI scores might be the low granularity of the visual score. Especially border cases, which are close to the threshold of 50%, can be justifiably assigned a score of 1 or 2, which has a big influence on the final score, while for QDP the differences would be marginal. Notably, overcoming these known shortcomings of the visual perfusion score was one motivation for the development of QDP.

None of the quantitative perfusion parameters correlated with LCI. Since LCI measurements were available only for a small sample size with only three LCI values in the group of patients with BAD and 21 patients without BAD, the statistical meaning of the calculated correlation is very limited. However, while a correlation of LCI with the MRI perfusion score was previously shown, the same study also identified subjects with LCI values below the upper limit of normal but marked perfusion abnormalities [11]. It may be discussed that perfusion MRI, including its quantitative metrics, may be more sensitive to functional lung abnormalities not captured by the LCI subjects with mild CF lung disease. Considering the population for which LCI was available for the present study (13.2 ± 3.8 years old; Perfusion Score: 5.3 ± 2.4), this may have led to a missing correlation (Table 2).

As a potential confounder, age might affect the results on quantitative perfusion metrics. Wehrum et al could show that in older patients (age range 60–80 years), pulmonary hemodynamics were altered, e.g., with lower peak flow volume, peak and mean flow velocities in older patients compared to younger patients (age range 20–39 years) [31]. Also, Levin et al found an increase of relative dispersion as a global index of spatial heterogeneity of PBF with age and also with height [32]. However, data including the whole age range of our study, and especially on children and adolescents, are entirely lacking.

By quantifying the timing of the peak signal of pulmonary enhancement separately inside normally perfused lung and perfusion defects using perfusion delay, we now demonstrate that inside perfusion defects, a delayed enhancement pattern can be quantitatively assessed (Figs. 1 and 2, Table 3).

The increased perfusion delay in perfusion defects confirms our assumption that perfusion abnormalities related to hypoxic pulmonary vasoconstriction may unmask the otherwise concealed enhancement peak coming from bronchial arterial inflow. There was also a widened peak with a high number of voxels showing an even later enhancement at histogram analysis (Fig. 2). For voxels with a very long perfusion delay, e.g., > 20 s (Figs. 2 and 4), this approach cannot separate an early recirculation of contrast through the pulmonary vascular bed from the pulmonary arteries vs. a very late inflow through bronchial arteries. This, however, has a minor implication for the determination of the peak at much shorter perfusion delay values. The regulation of bronchial arterial perfusion differs from that of pulmonary arterial perfusion, and increased flow through bronchial arteries in CF is probably driven by regional hypoxia and chronic inflammation [4, 8, 13]. It is known that Gd-based contrast is not an exclusively intravascular contrast agent, and leakage into the pulmonary interstitium could potentially affect the time course of signal as measured in this study. However, this effect happens on the order of minutes and should be negligible for the timescales of perfusion delay (~ 6 s) we examined here [33, 34]. Also, to our knowledge, there is no known potential for collateral inflow from neighboring lung tissue into perfusion defects.

To the best of our knowledge, no previous study aimed at quantitatively assessing bronchial arterial inflow with perfusion MRI, especially in the condition of BAD.

Additionally, we compared the perfusion parameters in patients with and without visible BAD. As in our previous report, patients with BAD were older, had worse lung function and higher MRI morphology, MRI perfusion, and MRI global scores than patients without (Table 1) [13]. The present study further showed higher QDP and lower PBF in the lungs of CF patients with BAD (Table 1). We selectively compared the perfusion parameters inside perfusion defects in patients with BAD to perfusion defects in patients without BAD. Interestingly, we found that perfusion delay was even longer in patients with BAD, which can also be appreciated from a further shift to later enhancement in the histogram (Figs. 3 and 4, and Table 3). This finding is somewhat counterintuitive to the idea that dilated bronchial arteries should deliver a higher inflow than in patients with non-dilated bronchial arteries. However, the rather tortuous course found in dilated bronchial arteries will certainly alter hemodynamics and laminar flow, potentially delaying delivery [35]. However, MTT was markedly shorter, and the histogram peak was narrower in defects in patients with BAD compared to patients without (Fig. 4, Table 3). This indicates—irrespective of the bolus arrival time—a faster throughput of the contrast bolus through defect areas within a shorter timeframe. In summary of these findings, one interpretation might be that bronchial arteries are dilated but can only partially compensate for intrapulmonary mechanisms that are impairing blood delivery rather than a ‘hypertrophy’ which would deliver a quantitatively increased systemic blood inflow. PBF alone only describes the blood flow under the assumption that contrast enhancement is entirely caused by the same contrast bolus described by the AIF. Accordingly, it is not well suited to separate contributions of pulmonary and bronchial arteries, since the latter both introduce a very similar time-curve of enhancement and are fairly close in time compared to the width of the bolus.

The strength of the cross-correlation between the AIF and the signal in a voxel, i.e., the arterial correlation, may be another promising approach to distinguish between patients with and without BAD. Patients with BAD had more voxels with lower arterial correlation especially inside the perfusion defects. Using histograms for arterial correlation, a significantly wider peak, which was shifted toward lower arterial correlations, was observed for the defect areas in BAD patients compared to patients without BAD (Fig. 4). The flow through the bronchial arteries generates a shunt volume from the systemic into the pulmonary circulation [36]. This means that the measured signal in DCE-MRI is a superposition of the signal from the pulmonary and systemic flow. A dilatation of bronchial arteries leads to higher blood flow and an increased shunt volume, which means that the proportion of the systemic signal to the pulmonary signal is increased. The lower arterial correlation in patients with BAD compared to patients without BAD might be a way to quantify this change in contribution of the systemic flow to the pulmonary flow and therefore the observed signal. Bronchial to pulmonary artery shunts are the anatomical basis for blood delivery to the pulmonary vascular bed from bronchial arteries, which thus allowed us to detect the signal from inside the lungs. Normally these anastomoses have no function and cannot be visualized with any clinical imaging technique. In chronic lung disease, they can be opened and modulate pressure of the pulmonary arterioles with systemic pressure [37, 38]. Bidirectional precapillary bronchopulmonary arterial anastomoses may exist from the level of the lobar bronchi. In cases of impaired inflow from either side, these shunts may open within 30 min [39]. At direct angiography, these shunts can be seen during bronchial artery embolization for hemoptysis in cystic fibrosis. Habert et al could also show particle shunting to the pulmonary vessels after bronchial artery embolization in cystic fibrosis, indicating a high prevalence of these anastomoses [40]. Using phase-contrast MRI, Ley et al could show an increased bronchosystemic shunt, indicating an increased flow through bronchial arteries, but which could not be depicted directly in this work [41]. As a future perspective, perfusion MRI could be complemented by phase-contrast MRI for the measurement of left and right cardiac outflow in order to calculate shunt ratios, which was previously shown to correlate with the CT-derived diameter of bronchial arteries in patients with chronic thromboembolic pulmonary hypertension [42].

This study has limitations. Breathing motion may affect our voxel-wise perfusion quantification pipeline. The voxel-wise perfusion quantification approach is vulnerable to breathing motion and may thus be distorted since our measurements were not exclusively acquired during static breath-holds. Further work to correct for breathing motion by separating it from dynamic contrast enhancement could noticeably improve this approach. Further, we did not separate by potential supply areas of the different bronchial arteries, since these are highly variable between individuals and supply areas are not well defined, and the relation between dilatation and a respective perfusion defect is not always clear [43].

In conclusion, our study provides further support for the use of QDP for quantitative assessment of pulmonary perfusion abnormalities as well as a potential endpoint for clinical trials in school-age to adult patients with CF. We were able to detect delayed perfusion in lung areas with perfusion defects and suggest perfusion delay and arterial correlation described in this work as promising novel markers to investigate the contribution of bronchial arteries to lung perfusion. Future studies should address the changes in these quantitative perfusion metrics under treatment with CFTR modulators in patients with and without bronchial artery dilatation.

## Supplementary information

ELECTRONIC SUPPLEMENTARY MATERIAL

Explanation Video

## Abbreviations

AIF: Arterial input function
BAD: Bronchial artery dilatation
CF: Cystic fibrosis
CFTRm: Cystic fibrosis transmembrane conductance regulator modulator
COPD: Chronic obstructive pulmonary disease
DCE-MRI: Dynamic contrast-enhanced MRI
ETI: Elexacaftor/tezacaftor/ivacaftor
LCI: Lung clearance index
MTT: Mean transit time
PBF: Pulmonary blood flow
PBV: Pulmonary blood volume
ppFEV1: Forced expiratory volume in 1 s in percent predicted
QDP: Perfusion defects in percent
R(t): Residual function
