# Supplementary material for: Quantitative MRI detects delayed perfusion and impact of bronchial artery dilatation on pulmonary circulation in patients with cystic fibrosis
Source: Eur Radiol. 2025 Apr 16;35(10):6217–28. doi: 10.1007/s00330-025-11589-y (PMC12417259; doi:10.1007/s00330-025-11589-y)
Supplement: Supplementary file 1 — ELECTRONIC SUPPLEMENTARY MATERIAL [file 330_2025_11589_MOESM1_ESM.pdf]

Quantitative MRI Detects Delayed Perfusion and Impact of Bronchial Artery Dilatation on Pulmonary Circulation in Patients with Cystic Fibrosis

ELECTRONIC SUPPLEMENTARY MATERIAL

Eur Radiol (2025) Leutz-Schmidt P, Grolig J, Wucherpfenning L, et al.

SUPPLEMENTAL METHODS

Subjects

The diagnosis of CF was based on newborn screening and/or clinical symptoms, confirmed by increased sweat Cl^- concentrations (≥60 mmol/l), *CFTR* mutation analysis, and in pancreatic sufficient patients with borderline sweat test results (Cl^- 30 - 60 mmol/l) by assessment of CFTR function in rectal biopsies (Table 1, Supplemental Table E1) [1]. Nitrogen (N_2) MBW (Exhalyzer D and Spiroware, Eco Medics AG, Duernten, Switzerland) was performed in 24 patients, and lung clearance index (LCI) values were corrected as described previously [2, 3]. Spirometry (MasterScreen Body, E. Jaeger, Hoechberg, Germany) was performed in 64 patients according to established standards [4]. Microbiology was determined by throat swabs in non-expectorating children, and sputum samples in expectorating children and adolescents on a quarterly basis. All patients received standard maintenance therapy as considered appropriate at the time of examination and the European best practice guidelines [5].

Morpho-functional chest magnetic resonance imaging

Standardized chest MRI including contrast-enhanced four-dimensional (4D) perfusion imaging was acquired with three clinical 1.5T MR scanners (Magnetom Symphony, Avanto and Aera, Siemens Medical AG, Erlangen, Germany) as previously described, and MRI protocols were kept essentially constant over the study period [1, 2, 6–13]. Specifically, for 4D perfusion imaging a spoiled gradient echo sequence with view sharing (TWIST, Siemens Medical AG, Erlangen) with an in-plane resolution in coronal orientation of 1.2x1.7 mm in patients ≤6 years to 2.2x1.6 mm in school-age children and adults, and with 5.0 mm slice thickness was performed. A series of 20 to 30 whole-lung volumes at a temporal resolution of approx. 1.5 s^{-1} during injection of 0.1 mmol/kg body weight of a macrocyclic Gadolinium(Gd)-based contrast material (Dotarem, Guerbet, Villepinte, France; or Gadovist, Bayer Schering, Leverkusen, Germany) at 2 – 5 ml/s followed by a saline chaser were acquired. Adult patients additionally underwent a second perfusion acquisition at lower temporal but higher spatial resolution of

1.1x1.1 mm in plane and 1.6 mm slice thickness with a second contrast injection with contrast dose being split between the two injections (MR angiography) [14]. Acquisition was performed in two phases with an average of 16s acquisition time each and a fixed delay of 10s in between to allow for short respiration in between, in order to achieve pulmonary and systemic arterial enhancement.

Chest magnetic resonance imaging scoring system

Structural and functional lung abnormalities were assessed using the dedicated MRI score by a reader with more than twelve years of experience in chest MRI (MOW) as previously described [2, 5–11, 13]. The MRI score comprises a MRI morphology score with subscores for structural lung changes and mucus plugging, and a MRI perfusion score. These are summarized in the MRI global score. The extent of abnormalities for each subscore was rated by lobe with 0 = no abnormality, 1 = <50% of the lobe involved, or 2 = ≥50% of the lobe involved, amounting to a theoretical maximum score of 60 [6]. In addition to the MRI score, the presence of BAD was assessed by this reader in consensus with a radiologist with more than five years of experience in chest MRI (PLS). Both readers were blinded for all clinical data. The timepoint with maximal enhancement of the aorta was selected from the full 4D perfusion MRI dataset and all visible vessels originating from the proximal aorta at the level of the carina were inspected. Because the in-plane resolution of the MRI technique precludes the detection of normal bronchial arteries that are smaller than approx. 2 mm, each artery in a typical anatomical position for a bronchial artery with a tortuous and unequivocal course along the main stem bronchi into the right or left lung hilus were rated as a positive finding for BAD (Figure E1).

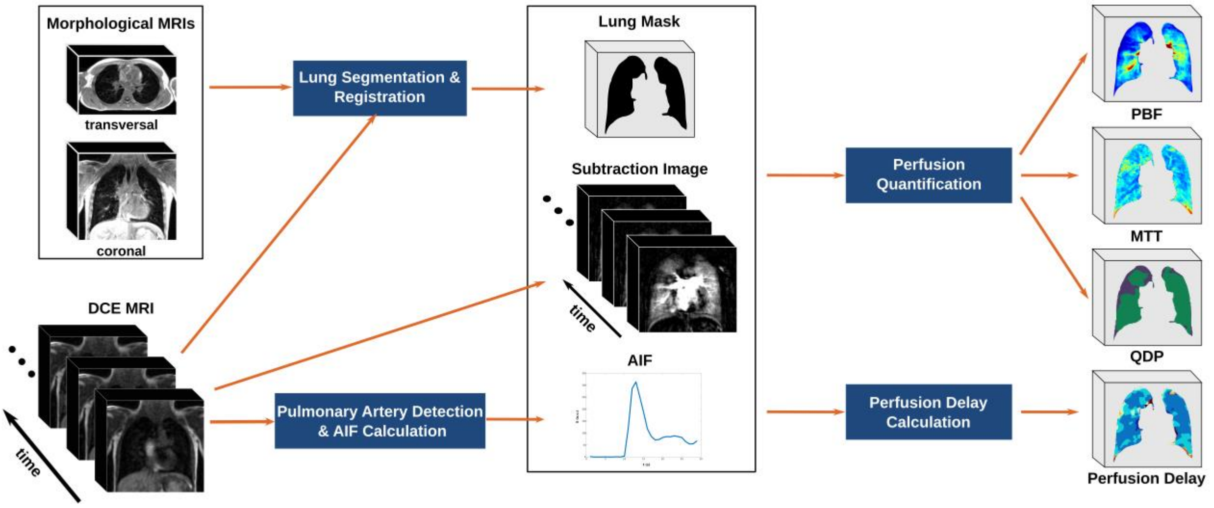

Supplemental Figure E1. Overview of the perfusion quantification concept. Lung masks are obtained from segmenting the morphological images. The quantitative perfusion maps are calculated from the DCE-MRI series.

Atypical variants originating from subclavian, thyrocervical or coronary arteries were not considered [15].

For quantitative assessment, the lung was automatically segmented on the morphological images and this segmentation registered onto the DCE-MRI images using a non-rigid registration with the use of elastix [16–18].

Multiple-breath washout

Multiple-breath washout (MBW) tests were performed using N_2 as marker gas. The measured LCI value was corrected based on the paper by Steinke et al. to correct for a spiroware software error [3]. The reported LCI values are LCI2.5% values, the values from the timepoint until the marker gas concentration is reduced below 2.5% of its starting concentration in the exhaled air.

MRI quantification pipeline

An overview of the applied perfusion quantification pipeline is shown in *Supplemental Figure E1*. For all patients the lung was automatically segmented in the morphological T1-weighted gradient echo (T1W-GRE) images. The obtained lung mask was then transferred into the DCE-MRI image space by registering the T1W-GRE images onto the DCE-MRI images using a combination of rigid-, affine- and BSpline-transformations with the registration tool elastix [19, 20]. From the DCE-MRI image I a subtraction image I_{Δ} is calculated by subtracting the mean of the first two pre-contrast timepoints**Error! Reference source not found.**The pulmonary a rtery is automatically detected and segmented in the DCE-MRI and the mask is used to obtain the arterial input function (AIF) C_a from I_{Δ}. Based on the indicator-dilution principles the residual function R(t) can be calculated by voxel-by-voxel deconvolution of I_{Δ} with the AIF using a truncated singular value decomposition approach:**Error! Reference source not found.**

$$C_a^{-1}(t) = V \cdot W \cdot U^T \quad (1)$$

Error! Reference source not found. Here V and U contain the left and right singular vectors o f matrix C and W contains the singular values of C. The residual function, the AIF and the subtraction image were afterwards used to calculate the pulmonary blood flow (PBF), the pulmonary blood volume (PBV) and the mean transit time (MTT): using equations **Error! R eference source not found.**

$$PBV = \frac{\int I_{\Delta}(t)\, dt}{\int C_a(t)\, dt} \quad (2)$$

Error! Reference source not found. Additionally, perfusion defects in percent (QDP) were q uantified based on the concept of Konietzke et al. by clustering the R(t) maps at the timepoint of maximum contrast enhancement using Otsu's method [19].

For the quantification of perfusion delay a low pass filter was applied to the subtraction image ($I_{\Delta} \rightarrow I_{\Delta,f}$). Then each voxel of $I_{\Delta,f}$ and the AIF were upsampled over time using cubic interpolation resulting in $I_{\Delta,f,i}$ and $C_{a,i}$. The cross-correlation between each voxel of $I_{\Delta,f,i}$ and a

moving window $\omega_{C_{a,i}}$ of the edge-padded $C_{a,i}$ was calculated:**Error! Reference source not f ound.**The perfusion delay for each voxel was finally defined as the position of the maximum of the calculated cross-correlation:**Error! Reference source not found.**The approach for the c alculation of the perfusion delay is visualized in Supplemental Figure E2.

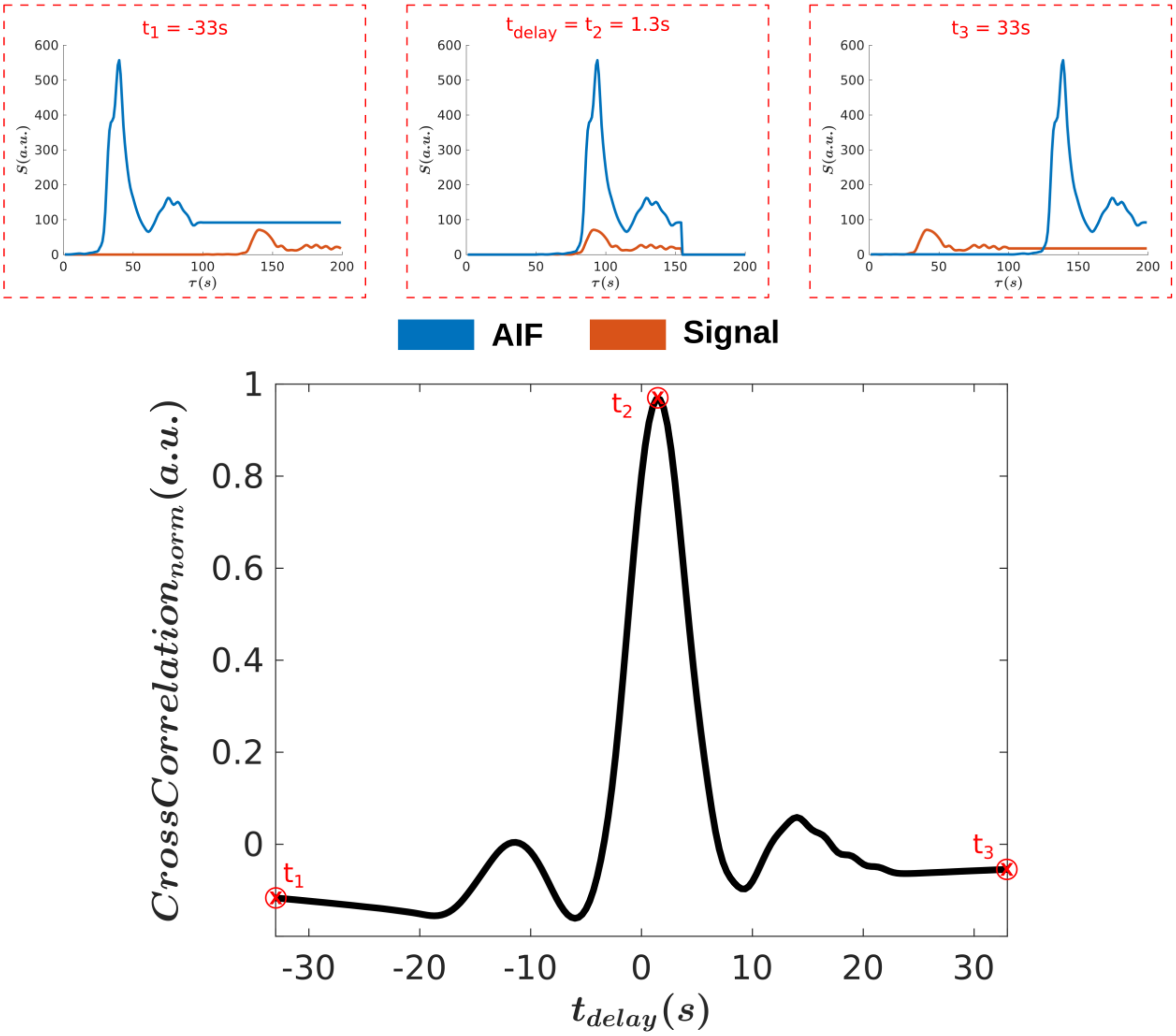

Supplemental Figure E2. Concept for the calculation of the perfusion delay. The signals of the edge padded AIF (blue) and the signal in a voxel (orange) are shown in the upper three plots for three different timepoints. The cross-correlation for each of these timepoints is marked in the cross-correlation plot on the bottom. The perfusion delay (t2 in this example) is the timepoint of highest cross-correlation.

Microbiology

Microbiology was determined by throat swabs in non-expectorating children and sputum samples in expectorating children, adolescents and adults on a quarterly basis. *Pseudomonas aeruginosa* infection status was defined according to the following criteria (E24-26): Patients were categorized as ‘without *Pseudomonas aeruginosa* infection’, when there was no growth of *Pseudomonas aeruginosa* in the previous twelve months and titers of precipitating antibodies against *Pseudomonas aeruginosa* antigens were negative (alkaline protease, elastase and exotoxin A; Mediagnost, Reutlingen, Germany), or as ‘intermittent *Pseudomonas aeruginosa* infection‘ when there was growth of *Pseudomonas aeruginosa* in <50% of cultures in the past twelve months and titers of precipitating antibodies against *Pseudomonas aeruginosa* antigens were negative, or as ‘chronic *Pseudomonas aeruginosa* infection’ when there was growth of *Pseudomonas aeruginosa* in ≥50% of cultures in the past twelve months, and / or increased levels (titer >1:1250) of two or more *Pseudomonas aeruginosa* antibodies, respectively.

Therapy regimes

All patients were followed at the CF center at the University Hospital Heidelberg and standard of care was provided according to the European Best Practice Guidelines including physiotherapy for airway clearance, inhalation with either isotonic or hypertonic saline starting at diagnosis, and substitution of pancreatic enzymes and fat-soluble vitamins in patients with pancreatic insufficiency [21, 22]. In case of worsening of respiratory symptoms or increasing MRI scores, inhaled dornase alfa was added. Detection of *Pseudomonas aeruginosa* led to an antibiotic treatment aiming at eradication. All other pathogens were generally treated with oral antibiotics if respiratory symptoms were present, however, no antibiotic prophylaxis was given [22, 23]. Only MRI examinations in stable clinical condition were included in the final analysis (Supplemental Figure E1) [23].

SUPPLEMENTAL REFERENCES

1. Graeber SY, Vitzthum C, Pallenberg ST, et al (2022) Effects of Elexacaftor/Tezacaftor/Ivacaftor Therapy on CFTR Function in Patients with Cystic Fibrosis and One or Two F508del Alleles. Am J Respir Crit Care Med 205:540–549. https://doi.org/10.1164/rccm.202110-2249OC
2. Stahl M, Wielpütz MO, Graeber SY, et al (2017) Comparison of Lung Clearance Index and Magnetic Resonance Imaging for Assessment of Lung Disease in Children with Cystic Fibrosis. Am J Respir Crit Care Med 195:349–359. https://doi.org/10.1164/rccm.201604-0893OC
3. Steinke E, Wielpütz MO, Joachim C, et al (2024) Reanalysis of N2-lung clearance index and the comparison to SF6-lung clearance index and magnetic resonance imaging. Journal of Cystic Fibrosis 23:150–154. https://doi.org/10.1016/j.jcf.2023.05.006
4. Mall MA, Hartl D (2014) CFTR: cystic fibrosis and beyond. Eur Respir J 44:1042–1054. https://doi.org/10.1183/09031936.00228013
5. Wielpütz MO, Eichinger M, Biederer J, et al (2016) Imaging of Cystic Fibrosis Lung Disease and Clinical Interpretation. Rofo 188:834–845. https://doi.org/10.1055/s-0042-104936
6. Eichinger M, Optazaite D-E, Kopp-Schneider A, et al (2012) Morphologic and functional scoring of cystic fibrosis lung disease using MRI. European Journal of Radiology 81:1321–1329. https://doi.org/10.1016/j.ejrad.2011.02.045
7. Wielpütz MO, Puderbach M, Kopp-Schneider A, et al (2014) Magnetic Resonance Imaging Detects Changes in Structure and Perfusion, and Response to Therapy in Early Cystic Fibrosis Lung Disease. Am J Respir Crit Care Med 189:956–965. https://doi.org/10.1164/rccm.201309-1659OC
8. Wielpütz MO, von Stackelberg O, Stahl M, et al (2018) Multicentre standardisation of chest MRI as radiation-free outcome measure of lung disease in young children with cystic fibrosis. Journal of Cystic Fibrosis 17:518–527. https://doi.org/10.1016/j.jcf.2018.05.003
9. Wielpütz MO, Eichinger M, Wege S, et al (2019) Midterm Reproducibility of Chest Magnetic Resonance Imaging in Adults with Clinically Stable Cystic Fibrosis and Chronic Obstructive Pulmonary Disease. Am J Respir Crit Care Med 200:103–107. https://doi.org/10.1164/rccm.201812-2356LE
10. Triphan SMF, Stahl M, Jobst BJ, et al (2020) Echo Time-Dependence of Observed Lung T1 in Patients With Cystic Fibrosis and Correlation With Clinical Metrics. J Magn Reson Imaging 52:1645–1654. https://doi.org/10.1002/jmri.27271
11. Stahl M, Steinke E, Graeber SY, et al (2021) Magnetic Resonance Imaging Detects Progression of Lung Disease and Impact of Newborn Screening in Preschool Children with Cystic Fibrosis. Am J Respir Crit Care Med 204:943–953. https://doi.org/10.1164/rccm.202102-0278OC
12. Wucherpfennig L, Triphan SMF, Wege S, et al (2022) Magnetic resonance imaging detects improvements of pulmonary and paranasal sinus abnormalities in response to elexacaftor/tezacaftor/ivacaftor therapy in adults with cystic fibrosis. J Cyst Fibros 21:1053–1060. https://doi.org/10.1016/j.jcf.2022.03.011
13. Stahl M, Wielpütz MO, Ricklefs I, et al (2019) Preventive Inhalation of Hypertonic Saline in Infants with Cystic Fibrosis (PRESIS). A Randomized, Double-Blind, Controlled Study. Am J Respir Crit Care Med 199:1238–1248. https://doi.org/10.1164/rccm.201807-1203OC
14. Wucherpfennig L, Triphan SM, Weinheimer O, et al (2023) Reproducibility of pulmonary magnetic resonance angiography in adults with muco-obstructive pulmonary disease. Acta Radiol 64:1038–1046. https://doi.org/10.1177/02841851221111486
15. Monroe EJ, Pierce DB, Ingraham CR, et al (2018) An Interventionalist's Guide to Hemoptysis in Cystic Fibrosis. Radiographics 38:624–641. https://doi.org/10.1148/rg.2018170122
16. Kohlmann P, Strehlow J, Jobst B, et al (2015) Automatic lung segmentation method for MRI-based lung perfusion studies of patients with chronic obstructive pulmonary disease. Int J CARS 10:403–417. https://doi.org/10.1007/s11548-014-1090-0

17. Klein S, Staring M, Murphy K, et al (2010) elastix: a toolbox for intensity-based medical image registration. IEEE Trans Med Imaging 29:196–205. https://doi.org/10.1109/TMI.2009.2035616
18. Shamonin DP, Bron EE, Lelieveldt BPF, et al (2014) Fast Parallel Image Registration on CPU and GPU for Diagnostic Classification of Alzheimer’s Disease. Front Neuroinform 7:. https://doi.org/10.3389/fninf.2013.00050
19. Konietzke P, Brunner C, Konietzke M, et al (2023) GOLD stage-specific phenotyping of emphysema and airway disease using quantitative computed tomography. Front Med (Lausanne) 10:1184784. https://doi.org/10.3389/fmed.2023.1184784
20. Schiwek M, Triphan SMF, Biederer J, et al (2022) Quantification of pulmonary perfusion abnormalities using DCE-MRI in COPD: comparison with quantitative CT and pulmonary function. Eur Radiol 32:1879–1890. https://doi.org/10.1007/s00330-021-08229-6
21. Smyth AR, Bell SC, Bojcin S, et al (2014) European Cystic Fibrosis Society Standards of Care: Best Practice guidelines. J Cyst Fibros 13 Suppl 1:S23-42. https://doi.org/10.1016/j.jcf.2014.03.010
22. Castellani C, Duff AJA, Bell SC, et al (2018) ECFS best practice guidelines: the 2018 revision. J Cyst Fibros 17:153–178. https://doi.org/10.1016/j.jcf.2018.02.006
23. Rosenfeld M, Ratjen F, Brumback L, et al (2012) Inhaled hypertonic saline in infants and children younger than 6 years with cystic fibrosis: the ISIS randomized controlled trial. JAMA 307:2269–2277. https://doi.org/10.1001/jama.2012.5214

SUPPLEMENTAL TABLES

**Pancreatic insufficiency**		**Pancreatic sufficiency**	
***CFTR* genotype**	**Subjects, n**	***CFTR* genotype**	**Subjects, n**
**Total**	**66**	**Total**	**8**
F508del/F508del	34	F508del/M1101K	1
F508del/621+1G>T	3	F508del/R347P	1
F508del/G542X	2	F508del/R117H	1
F508del/N1303K	2	F508del/R553X	1
F508del/Q220X	2	F508del/3849+10kb C>T	1
F508del/CFTRdele2,3	2	F508del/5T-Allel	1
F508del/2183AA>G	2	I507del/3849+10kb C>T	1
F508del/E60X	1	R553X/3849+10KbC>T	1
F508del/G551D	1		
F508del/I507del	1		
F508del/M1101K	1		
F508del/T1299I	1		
F508del/Q39X	1		
F508del/Q552X	1		
F508del/R1066H	1		
F508del/R347P	1		
F508del/V520F	1		
F508del/CFTRdele17	1		
F508del/1341+1G>A	1		
F508del/2143delT	1		
F508del/3821delT	1		
F508del/3905insT	1		
F508del/909delT	1		
R347P/R347P	1		
W1282X/2991del321	1		
1717-1G->A/1717-1G->A	1		

Supplemental Table E1. Summary of *cystic fibrosis transmembrane conductance regulator* (*CFTR)* genotypes in patients with cystic fibrosis grouped according to pancreatic functional status. 1 Genotype is missing.

	Age Range 6 – 17yrs	
	**No BAD**	**BAD**
**n**	26	13
**Age (y) (range)**	12.5±3.5 (6-17)	13.1±3.1 (7-17)
**Male / female**	14/12	7/6
**Height (cm)**	152.8±20.4	155.7±14.2
Height, SDS†	0.0±1.1	-0.1±1.4
**Weight (kg)**	43.4±17.5	42.7±10.0
Weight, SDS†	-0.5±0.9	-0.8±1.0
**BMI, (kg/m2) ♦**	17.6±3.2	17.4±2.4
BMI, SDS†	-0.7±0.8	-0.9±0.9
**Spirometry, n**	26	11
ppFEV$_1$	84.2±16.2	66.8±15.7***
**MBW, n**	20	3
LCI2%	8.3±2.2	10.7±2.8
**MRI morphology score**	8.8±5.7	13.2±4.6**
**MRI perfusion score**	4.7±2.4	6.5±1.5*
**MRI global score**	13.6±7.5	19.8±5.7***
**QDP (%)**	17.7±10.9	25.9±15.7
**PBF (ml/100ml*min)**	179.7±78.6	137.4±65.9
**MTT (s)**	6.1±2.2	5.9±1.9
**Perfusion delay (s)**	5.1±5.8	4.2±1.7
**Arterial correlation**	0.91±0.06	0.90±0.06

Supplemental Table E2. Age-matched patient characteristics and quantitative perfusion parameters for the two age ranges of 6-17 yrs and 12-17 yrs.

BMI = body mass index, ppFEV1 = forced expiratory volume in 1 s in percent predicted, LCI = lung clearance index, MBW = Multiple-breath washout, QDP = quantitative defects in percent, PBF = pulmonary blood flow, MTT = mean transit time. Data presented as percentage (proportion) or mean ± SD. **P*<0.05, ***P*<0.01, and ****P*<0.001 vs. no BAD, respectively. †SDS given for patients ≤18 y only, ♦BMI given for patients >18 yrs only.
